# Growth Performances and Nutritional Values of *Tenebrio molitor* Larvae: Influence of Different Agro-Industrial By-Product Diets

**DOI:** 10.3390/foods15020393

**Published:** 2026-01-22

**Authors:** Giuseppe Serra, Francesco Corrias, Mattia Casula, Maria Leonarda Fadda, Stefano Arrizza, Massimo Milia, Nicola Arru, Alberto Angioni

**Affiliations:** 1 Institute of BioEconomy, National Research Council, Trav. La Crucca, 3, 07100 Sassari, Italy; giuseppe.serra@cnr.it (G.S.); marialeonarda.fadda@cnr.it (M.L.F.); stefano.arrizza@cnr.it (S.A.); 2 Food Toxicology Unit, Department of Life and Environmental Science, University of Cagliari, University Campus of Monserrato, SS 554, 09042 Cagliari, Italy; m.milia24@studenti.unica.it (M.M.); nicola.arru@unica.it (N.A.); aangioni@unica.it (A.A.)

**Keywords:** yellow mealworm, feed macro-composition, insect macro-composition, larval growth, feed conversion indices

## Abstract

Intensive livestock and aquaculture systems require high-quality feeds with the correct nutritional composition. The decrease in wild fish proteins has led to demands within the feed supply chain for new alternatives to fulfil the growing demand for protein. In this context, edible insects like the yellow mealworm (*Tenebrio molitor*) have the greatest potential to become a valid alternative source of proteins. This study evaluated the growth performance and nutritional profile of yellow mealworm larvae reared under laboratory conditions on eight different agro-industrial by-products: wheat middling, durum wheat bran, rice bran, hemp cake, thistle cake, dried brewer’s spent grains, dried tomato pomace, and dried distilled grape marc. The quantitative and qualitative impacts of rearing substrates on larvae were compared. The results showed that larvae adapt well to different substrates with different nutritional compositions, including the fibrous fraction. However, substrates affect larval growth feed conversion and larval macro composition. Hemp cake stood out for its superior nutritional value, as reflected by its high protein content and moderate NDF (Neutral Detergent Fiber) levels, which determine fast larval growth. On the contrary, imbalanced substrate lipid or carbohydrate content (rice bran), as well as the presence of potential antinutritional compounds (thistle cake), appeared to negatively affect growth performances.

## 1. Introduction

Globally, dietary proteins are mostly of plant origin, accounting for around 57% of the total. In Europe, however, the majority (55–60%) derive from animal sources [1]. The International Feed Industry Federation (IFIF) estimates that the global population will exceed 10 billion by 2050, necessitating a doubling of current animal protein production [2]. This increase in demand is expected to put further pressure on food production systems that are already under strain due to land and water scarcity, high dependency on inputs such as fertilizers, and significant environmental impact. The Food and Agriculture Organization (FAO) has emphasized that it is impossible to meet this rising demand, especially when relying on fish meal and fish oil used for intensive aquaculture feed [3]. The existing protein balance has a significant global environmental impact. Approximately three-quarters of agricultural land and two-thirds of greenhouse gas emissions are associated with animal-based foods [1]. In the European Union (EU), most of these protein sources, along with soybeans, are currently imported, which increases costs and has a further negative impact on the environment [4]. Consequently, the development of alternative protein sources for food and feed has become an increasingly urgent issue for Western countries.

In 2020, alternative proteins accounted for just 2% of the animal protein market, which was worth approximately 13 million tonnes [1]. The most prominent alternative protein sources are insects, microbial fermentation, algae, and cultured meat [1]. Thanks to their nutritional value, low competition for land resources, simple reproduction, and rapid growth, edible insects are considered a suitable alternative protein source for humans, livestock, and aquaculture [2]. In 2013, the FAO published an assessment of edible insects for use as food and feed [5]. More recently, the European Commission amended the EU’s feed ban regulation to authorize the use of processed animal proteins (PAPs) derived from insects in poultry and pig feed [6]. Production of insect feed is forecast to reach approximately 2.7 MT by 2030. According to the European Parliamentary Research Service (EPRS), 25–30% of fishmeal can be replaced by insect meals in aquaculture, whereas for poultry and pigs, this accounts for about 10% [1]. Although over 2000 species of edible insects have been identified, only a few are currently farmed on a large scale for food and feed [7].

Among these, the yellow mealworm (*Tenebrio molitor* L. Coleoptera, Tenebrionidae) is one of the candidates for replacing fishmeal in animal feed [8]. The larvae of *T. molitor* demonstrate excellent growth performance and contain high levels of protein and lipids, making them a valuable alternative to fish and soybean meals [9]. They also have a high content of essential amino acids and fatty acids [2]. *T. molitor* can be reared on various substrates and under different environmental conditions. Several authors have reported on trials breeding *T. molitor* on various substrates [10,11,12,13,14,15], followed by evaluations of the resulting larvae’s nutritional value [16,17], morphological parameters [18,19], or both [20,21].

This study aimed to evaluate the entire yellow mealworm (*Tenebrio molitor*) rearing process, including the proximate analysis of starting materials, the chemical evaluation of larvae, their growth performance, feed intake, and feed conversion efficiency. Furthermore, this study was the first to perform complete chemical analyses (ash, lipids, proteins, carbohydrates, NDF, hemicellulose, and cellulose) on both the residual post-rearing substrate and the larval faeces. These data were essential for fully evaluating the larvae’s selective feeding behaviour and adaptability to different substrates.

In accordance with the principles of the circular economy [22], mass-rearing *T. molitor* could represent an effective way to recover by-products and unusable waste derived from different agro-industrial supply chains and convert them into valuable resources for use as a component for fish feed.

## 2. Materials and Methods

### 2.1. Chemicals

Sulphuric acid (H_2_SO_4_, 96%), sodium hydroxide (NaOH 32%), copper sulphate (CuSO_4_), sodium sulphate (Na_2_SO_4_), potassium chloride (KCl), sulphuric acid (H_2_SO_4_, 0.5 N), hydrochloric acid (HCl, 37%), D(+)-glucose anhydrous, phenol, sodium borate decahydrate (Na_2_B_4_O_7_·10H_2_O), disodium EDTA (C_10_H_14_N_2_Na_2_O_8_), cetyl(trimethyl)ammonium bromide (CTAB—C_19_H_42_BrN), anhydrous hydrogen phosphate (Na_2_HPO_4_), neutral sodium lauryl sulphate (C_12_H_25_NaO_4_S), ethylene glycol monomethyl ether, and n-octanol (antifoaming agent) were purchased from Carlo Erba (Milan, Italy). Sodium hydroxide (NaOH) 0.5 N was purchased from Titolchimica S.p.A. (Villamarzana, Roma, Italy), whereas methanol (CH_3_OH) and chloroform (CHCl_3_) were purchased from Sigma Aldrich (Milan, Italy). Double-deionized water with a conductivity of less than 18.2 MΩ was obtained using a Milli-Q system (Millipore, Bedford, MA, USA).

### 2.2. Substrates

The feed substrates were selected from the main by-products of local agricultural and food processing chains, as well as from the milling industry and food and beverage manufacturing. Waste panels from cardoon seed oil extraction were also used. The raw materials were sourced from both producers and local factories.

Eight by-products were evaluated for their suitability as feed substrates for insect rearing (Table 1): wheat middling (WM), durum wheat bran (WB), rice bran (RB), hempseed cake (HC), thistle cake (TC), brewer’s spent grains (BSG), tomato pomace (TP), and distilled grape marc (DGM). Before use, the substrates BSG, TP, and DGM were oven-dried to reduce the water content. All substrates were separated from the coarse parts using a 2 mm sieve, and when necessary, they were ground with a mill to reduce them to diameters of less than 2 mm and stored at room temperature. Proximate analysis was performed on all samples.

### 2.3. *Tenebrio molitor* Farming

*T. molitor* larvae were obtained from a stock colony of adults reared on wheat bran and fresh vegetables as a water source, owned by the Institute of BioEconomy of the Italian National Research Council (CNR, Sassari, Italy).

Eggs were obtained from 7 days of oviposition of 1440 adults, 48 groups of 15 couples each, and reared separately in plastic pots (7.5 × 10.5 × 5.5 cm) containing 10 g of wheat bran under laboratory-scale production. After the oviposition, the adults and substrate were separated from the glued eggs at the bottom of the pot. Each pot was then randomly assigned to one of the eight experimental substrates (6 replicates per substrate). The eggs of each pot were counted, and 7 g of the feed substrate was added. The eggs and larvae were reared ad libitum, adding feed when necessary, in a controlled temperature and humidity environment (25 ± 2 °C, 60 ± 5% relative humidity) under 12:12 h of light–dark illumination. The water source (fresh carrots) was introduced in the third week after oviposition. The uneaten carrots were removed weekly. Larval weight was recorded from 4 weeks of rearing and subsequently at 14-day intervals to monitor growth. Frass was separated monthly or bimonthly using a 0.5 mm sieve. The trials were terminated when new pupae appeared consistently every day for over a week in each replicate (harvest stage). Larvae and pupae were then separated from residual unfed substrates and frass by sieving (2 mm and 0.5 mm). Subsequently, they were counted and weighed to determine the total final biomass and the mean larval and pupal weight.

Administered feed substrates (experimental dried substrate and fresh carrots), residual feed (unfed), live larvae and pupae, and frass were weighed using an analytical balance (XS BL 224 basic—Bormac srl., Modena, Italy). At the end of the experimental period, the larvae were separated from the leftover food and left without food for 24 h to give their guts time to clear out. Then, they were stored at −20 °C before being analyzed. The leftover food and frass were stored at 5 °C. The following parameters were used to evaluate larval growth performance: (i) average duration of larval development (number of days required to reach the harvest stage); (ii) larval mortality (% Dead individuals = N°Eggs_i_ − N°Larvae − Pupae_h_; i initial, h harvest stage); (iii) average pupation rate (% Pupae_alive_ = N° Larvae_alive_ vs. Pupae_alive_); (iv) average total final biomass; (v) larval weight; (vi) pupal weight.

Feed conversion efficiency was assessed using the feed conversion ratio (FCR) (1), the efficiency of conversion of ingested food (ECI) (2), the efficiency of conversion of digested food (ECD) (3), and the protein efficiency ratio (PER), calculated by dividing the weight gain of the larvae by the total protein fed [23,24,25]:

(1) $$FCR=\frac{feed\;ingested\;(g)}{larval\;weight\;gain\;(g)}$$

(2) $$ECI=\frac{larval\;weight\;gain\;(g)}{feed\;ingested\;(g)}$$

(3) $$ECD=\frac{larval\;weight\;gain\;(g)}{feed\;ingested\left(g\right)-frass\;(g)}$$

The carrot consumption ratio (FCRC) (4) per larval biomass unit was determined as follows:

(4) $$FCRc=\frac{carrot\;ingested\;(g)}{larval\;weight\;gain\;(g)}$$

The amount of feed ingested was calculated by subtracting the amount of feed recovered from the amount of feed given. The amount of carrot ingested was calculated by subtracting the amount of carrot recovered from the amount of carrot given. The larval weight gain was equal to the larval weight at harvest time due to the negligible weight of the eggs at the beginning of the trial.

The frass ratio (FP) (5) was calculated as the total frass collected and the total weight of the larvae:

(5) $$FP=\frac{total\;frass\;(g)}{larval\;weight\;gain\;(g)}$$

### 2.4. Proximate Composition

Chemical analyses were conducted on representative batches of mature larvae, raw feed substrates, and residual materials (including frass) separated from the larvae and the pupae at the end of the trial.

#### 2.4.1. Moisture and Ash

An aliquot of the homogenized sample (0.5 g) was weighed in a porcelain crucible and dried at 105 °C in a thermostatic oven (ISCO, series 9000, Milan, Italy) until a constant weight was achieved (approximately 24 h). The samples were stored in a desiccator at ambient temperature and weighed to determine their moisture content. The same samples were then placed in a muffle furnace at 550 °C for five hours to determine the total ash content. Moisture and ash content were expressed as g/100 g.

#### 2.4.2. Total Lipids

Total lipids were evaluated according to the modified Folch method. Briefly, 0.2 g of the homogenized sample was weighed into a 15 mL Falcon tube, along with 1.5 mL of MeOH and 3 mL of CHCl_3_. The mixture was agitated for 1 min and then stirred for 30 min using a rotary shaker Falc F205 G (Treviglio, Bergamo, Italy). Subsequently, 1.0 mL of KCl solution (0.2 M) was added to facilitate phase separation. The tube was then centrifuged at 3154× *g* for 15 min. One mL of the organic CHCl_3_ phase was placed in a pre-weighed glass vial and dried under a gentle nitrogen stream. The remaining lipid fraction was expressed in g/100 g.

#### 2.4.3. Total Carbohydrates

Total carbohydrate analysis was performed by following the method proposed by Dubois et al. [26]. Briefly, 20 mg of the sample was placed in a 15 mL Falcon tube with 5 mL of HCl (1 M) and heated at 100 °C for 2 h to hydrolyze carbohydrates and release glucose. The hydrolyzed extract (1 mL) was poured into a 15 mL glass tube plus 1 mL of phenol (5% in water, *w*/*v*) and 5 mL of H_2_SO_4_ (96%), shaken gently for a minute at room temperature, and left for 30 min in the dark to rest. The samples were then analyzed using a Cary 50 UV-Visible Spectrophotometer (Varian Inc., Agilent, Milan, Italy) at 488 nm vs. a control blank (1 mL H_2_O, 1 mL phenol 5%, and 5 mL H_2_SO_4_). Total carbohydrates were expressed as g/100 g of D-glucose equivalent using a five-point calibration curve with a coefficient of determination (r^2^) ≥ 0.997.

#### 2.4.4. Total Proteins and Total N Content

Total protein and N content were determined using the Kjeldahl digestion method [27]. Briefly, 0.5 g of the sample was weighed into a digestion flask and processed using a BUCHI Speed-Digester K-424 (BUCHI, Cornaredo, Milan, Italy) according to the manufacturer’s instructions. Subsequently, 20 mL of H_2_SO_4_ (96%), 0.5 g of Na_2_SO_4_, and a small amount of CuSO_4_ were added to the digestion flask. After mineralization, the solution was left to cool, and 50 mL of Milli-Q water was added. Once the solution had turned light blue, the digestion flask was transferred to the BUCHI K-314 distillation unit (BUCHI, Cornaredo, Milan, Italy). A NaOH solution (32%, *w*/*v*) was then added to the sample to convert NH_4_^+^ into NH_3_. The released NH_3_ was distilled and captured in a receiving flask containing 10 mL of H_2_SO_4_ (0.5 N) and a few drops of methyl red as an indicator, until 150 mL of the distillate was collected. After distillation, a quantitative analysis was performed using acid–base titration with 0.5 N NaOH. Total N (6) and protein (7) content were calculated using the following equations and expressed as g/100 g:

(6) $$N=\frac{\left(a-b\right)\times c\times 100}{Sample\;weight\;(g)}$$

(7) $$Protein=\frac{\left(a-b\right)\times c\times 100\times K}{Sample\;weight\;(g)}$$

a: mL of H_2_SO_4_ (0.5 N)
b: mL of NaOH (0.5 N)
c: nitrogen conversion factor (0.007 g N/mL) for H_2_SO_4_ (0.5 N)
K: nitrogen-to-protein conversion factor (6.25 for feed substrates, 4.76 for mealworm larvae [26])
Sample weight: weight (g) of the sample analyzed.

Total N was used for larval growth and frass, whereas total protein was used for feed substrate and larval composition [28].

#### 2.4.5. Fiber

Neutral detergent fibre (NDF) and acid detergent fibre (ADF) were evaluated according to Van Soest et al. using a FIWE Advance Automatic Fiber Analyzer (VELP Scientific, Usmate, MB, Italy) [29]. One gram of sample was digested for 2 h with 100 mL of a neutral detergent solution (sodium lauryl sulphate 30 g/L, disodium EDTA 18.61 g/L, sodium borate decahydrate 6.18 g/L, anhydrous hydrogen phosphate 4.56 g/L, ethylene glycol monomethyl ether 10 mL, with pH between 6.9–7.1) to obtain the insoluble fraction. The residue (NDF), which contained hemicellulose, cellulose, and lignin, was first washed with boiling water and then with acetone. It was then dried at 105 °C for 8 h and weighed. The resulting sample was refluxed with 100 mL of H_2_SO_4_ (49 g/L) and CTAB (20 g/L) for 1 h to solubilize the hemicellulose fraction. Following filtration, the residue was washed with boiling water, dried at 105 °C for 8 h, and weighed. This fraction (ADF) consists of cellulose and lignin. Hemicellulose was calculated by subtracting the ADF value from the NDF value. The results were expressed as g/100 g.

### 2.5. Statistical Analysis

Statistical analyses were performed using GraphPad Prism software (version 10.4.1). Mean differences were assessed using multiple *t*-tests, with *p*-values adjusted using either the Bonferroni–Dunn correction or the Benjamini–Krieger–Yekutieli false discovery rate (adaptive FDR) procedure [30]. A significance threshold of *p* < 0.05 was applied, and corrected results are reported in Supplementary tables (Tables S1–S3). Pearson’s correlation coefficient was used to calculate the effect of the variation of one variable as the other varied.

#### The Rationale for the Selection of Specific Statistical Approaches

Conducting multiple statistical tests simultaneously increases the probability of obtaining false-positive results. In this study, a significance threshold of *p* < 0.05 was applied to individual tests. To account for multiple comparisons, two different correction approaches were considered.

The Bonferroni–Dunn procedure controls the family-wise error rate (FWER), i.e., the probability of committing at least one Type I error across the set of tests. However, this approach is highly conservative and may substantially reduce statistical power when a large number of comparisons are performed, increasing the risk of false negatives [30].

In contrast, the Benjamini–Krieger–Yekutieli (BKY) adaptive false discovery rate (FDR) correction controls the expected proportion of false positives among the results declared significant and is therefore less conservative. This method allows the identification of a greater number of potentially relevant differences without compromising overall statistical validity and is particularly suitable for exploratory analyses involving multiple comparisons [30].

Accordingly, BKY-FDR–adjusted values were used for data interpretation in this section, whereas Bonferroni–Dunn–corrected results are reported in the Supplementary Tables (Tables S1–S3) for comparison. Overall, the analysis revealed minor but statistically significant differences in larval development, chemical composition, remaining feed, and frass among the different substrate trials.

## 3. Results

### 3.1. Proximate Analysis of Substrates

The raw substrates selected for the trials varied in their proximate chemical composition (Table 2). The moisture content ranged from 10.09 ± 7.47% g/100 g (WM) to 5.09 ± 2.84% g/100 g (TC). RB had the highest ash content (8.19 ± 2.29% g/100 g), and BSG had the lowest (3.36 ± 4.21% g/100 g). Total lipids ranged from 5.22 ± 5.07% g/100 g (DGM) to 15.43 ± 7.07 g/100 g (RB), and total proteins ranged from 27.40 ± 4.86% g/100 g (HC) to 7.06 ± 12.38% g/100 g (RB). Carbohydrates were higher in the substrate from the milling industry (RB, WM, and WB), averaging 35.60 g/100 g, compared to the other substrates, which ranged from 14.50 ± 3.95% g/100 g (TC) to 5.68 ± 12.35% g/100 g (BSG). Conversely, TP, BSG, TC, and DGM showed higher NDF concentrations. The proximate composition of HC, WB, and BSG was similar to that reported by Lienhard et al., with only minor differences related to the origin of the raw materials [21]. The TP, WM, RB, TC, and DGM compositions were consistent with the variability ranges in the literature [31,32,33,34,35].

### 3.2. Larval Chemical Composition

The analysis of the macro-compounds in the larvae showed only minor statistical deviations among substrates (Table 3 and Table S1). The moisture content was generally constant, with a very slight tendency to increase in larvae reared on lower-quality feed. The average moisture content ranged from 58.83 g/100 g (WB) to 65.66 g/100 g (TC). The ash averaged 1.17 g/100 g, except for DGM (1.66 g/100 g) and RB (1.46 g/100 g), which showed the highest values. DGM had the highest lipid content at 16.70 g/100 g, which was statistically different from that of all the other substrates. The protein fraction represents the main component among macronutrients; its levels were stable across the various substrates, ranging from 17.91 g/100 g (RB) to 19.86 g/100 g (HC), but with no statistical deviation. Carbohydrate content remained low across all groups, with minor fluctuations ranging from 6.17 g/100 g (WB) to 3.36 g/100 g (WM) (Table 3).

### 3.3. Chemical Analysis of Residual Feed (R) and Insect Frass (F)

To understand the larvae’s ability to use the different substrates, at the end of the rearing stage, the residual feeds for each substrate (acronym: substrateR) and insect frass (acronym: substrateF) were fully characterized and compared with the raw substrates.

**Figure 1:**
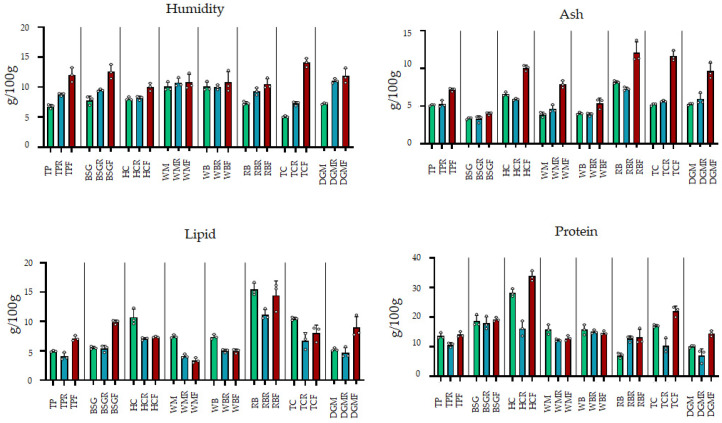
Chemical composition of raw substrates, remaining feed after consumption (R), and insect frass (F) (g/100 g). Detailed statistical comparisons for each parameter are provided in Supplementary Table S2.

Residual feed: Moisture values showed variation with respect to the raw substrates, although not always associated with a statistically significant variation (Figure 1, Table S2). TPR, BSGR, WBR, RBR, TCR, and DGMR had higher moisture content than the raw substrates, with values ranging from 16.4% for WBR to 52.0% for DGMR. The HCR and WMR showed the lowest moisture values. Ash levels between substrates and R matrices were, in most cases, not statistically different, except for HCR, which showed statistically lower values, and WBR, which showed higher levels. The total lipid and total protein content of all substrates was lower than that of the raw matrices. RBR showed controversial behaviour, showing a higher level of protein than the raw substrate (+80%).

Carbohydrates showed higher values in TPR and BSGR, lower in HCR, WBR, TCR, and DGMR, whereas no differences were detected in WMR and RBR.

**Figure 2:**
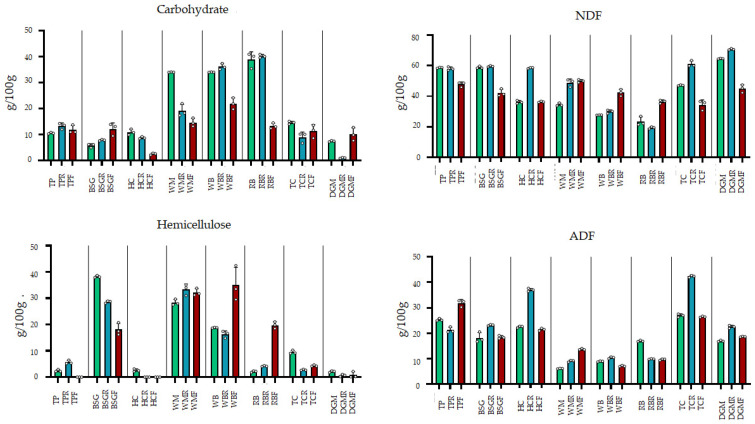
Carbohydrates and non-digestible fibrous fraction (NDF, hemicellulose, and ADF) of raw substrates, remaining feed after consumption (R), and insect frass (E) (g/100 g). Detailed statistical comparisons for each parameter are provided in Supplementary Table S2.

The fibre content (NDF) was consistently higher in the HCR, WBR, and TCR fractions, while RBR showed the lowest values. BSGR, WMR, TPR, and DGMR had values comparable to those of the original substrates (Figure 2). Carbohydrates and NDF showed a good correlation in the residual feed (r = −0.986).

Frass: All samples showed an increase in moisture content, except WBF. TCF showed the highest increase in moisture values (14.11 g/100 g, 175.4% of the raw substrate), whereas HCF showed the lowest increase, around 3.5% of the raw substrate. The increase in the other substrates averaged +67.02% (TPF, BSGF, and DGMF) and +33.88% (RBF and HCF), whereas WBF was +7.72%.

Frass moisture values correlated better with carrots than feed consumed (0.78 vs. 0.38). Therefore, TCF moisture values could be associated with the high carrot consumption ratio of larvae reared on this substrate. Ash levels were higher, with differences of up to 122.9% (TCF) (Figure 1, Table S2). Total lipids showed values below those of the raw substrates, except for TPF, BSGF, and DGMF. The total protein concentration was higher in RBF, DGMF, TCF, and HCF, whereas it decreased in WMF and WBF. Carbohydrates in frass were lower than in the raw substrate in six cases (TPF, HCF, WMF, WBF, RBF, and TCF) and higher in DGMF and BSGF, which showed the highest values (Table S2).

NDF showed an increase in WBF, TCF, and HCF, whereas TPF, BSGF, TCF, and DGMF decreased and HCF remained unchanged (Figure 2, Table S2). The NDF fraction consists mainly of the ADF. The frass samples belonging to larvae fed with TP and HC showed only traces of hemicellulose (Figure 2).

### 3.4. Larval Growth Performances

The initial average density was 2.98 ± 0.04 eggs per cm^2^. The number of *T. molitor* eggs/pot averaged 230 ± 5% (average ± RSD%). TC had the maximum number of eggs, accounting for 285 ± 1% (average ± RSD%).

There were significant differences in the time taken for larval development among the trials, with times ranging from 97.3 (WM) to 173.2 (DGM) days. WB, WM, HC, and BSG showed the fastest larval development time, with an average of 100 days (Table 4). At harvest time, DGM and TC larvae and pupae showed the lowest weight, statistically different from all other substrates, which averaged 0.133 ± 8.62 and 0.112 ± 11.14, respectively (g ± RSD%) (Table 4 and Table S3). RB, WM, HC, and WB showed the highest larval weight, averaging 0.140 ± 3.29 (g ± RSD%). Pupal weight did not have statistically significant differences among substrates. The lowest mortality rate was obtained by WB (4.27%), followed by HC (9.46%). WM, TC, and TP had medium mortality, whereas RB, DGM, and BSG showed the highest levels. No correlation was noticed between harvest time, mortality, and biomass.

In contrast, the trials showed a good correlation (r = 0.945) between harvest time and the rate of larvae/pupae; the lower the number of days, the greater the number of pupae compared to larvae. DGM, which had the highest harvest time, had the highest rate values, with a higher number of insects in the larval stage. The only substrate that did not demonstrate this behaviour was HC, which had the lowest rate of larvae and pupae (2.01 ± 54.03, average ± RSD%), with a medium harvest time (~100 days). The high variability in HC was related mainly to values below the average rate.

Concerning biomass, DGM showed lower values, together with RB, whereas WB, HC, and WM showed higher biomass values.

Pupation rates express the development of pupae from larvae; the highest values were detected in HC (36.9%), whereas DGM showed the lowest values (10.9%).

WB promoted optimal larval development, achieving a short developmental time of 98.7 days, the greatest final biomass (30.06 g), and average larval and pupal weights (0.135 g and 0.113 g, respectively) (Table 4). WM demonstrated comparable performance, with similar larval and pupal weights. However, the final biomass was slightly lower (25.23 g) due to higher mortality (19.86%) (Table 4).

### 3.5. Feed Conversion

**Figure 3:**
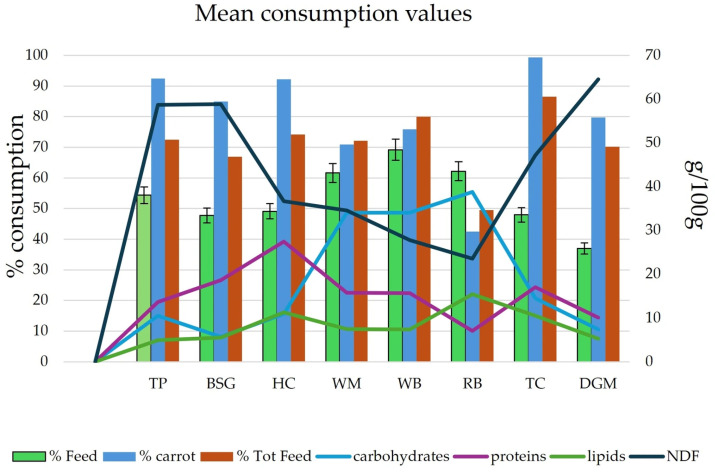
Mean consumption values of the eight substrates used for the development of *T. molitor* larvae and the chemical composition of the substrates.

Mean consumption values showed that *T. molitor* larvae preferred WM, WB, and RB, followed by TP, then TC, BSG, and HC. The lowest consumption rate was observed for DGM. When carrots were considered, the highest consumption rates were shown by TC, TP, HC, and BSG, followed by DGM, WB, and WM. RB showed the lowest consumption of carrots (Figure 3).

When considering the total amount of feed supplied to the larvae, TC demonstrated the highest consumption rate (86.5%), followed by WB, HC, WM, and TP, with an average consumption rate of 75.6%. BSG and DGM averaged 68.5%, whereas RB showed the lowest values, accounting for 49.4% of raw food consumption.

The feed conversion efficiency (FCR) represents the ratio between the feed ingested and the increase in larval weight. Lower FCR values reflect a more efficient conversion of feed into larval biomass (Table 5 and Table S1). HC showed the best average value (1.92), whereas the worst results were obtained with the RB substrate, accounting for an FCR of 3.23. FCRc showed the best ratio for TP, BSG, and RB, followed by HC, WM, and WB. The worst rates were for DGM and TC. FCRtot was strongly influenced by the carrots consumed and was similar to FCRc. The ECI calculation gives the efficiency of conversion of the ingested food. The results showed the best performances for HC, followed by WM, WB, TC, TP, and BSG. The worst results were detected for RB and DGM. The protein efficiency ratio (PER) showed differences among substrates that were not correlated with their protein content. This data agrees with other studies, which found no linear correlation between substrate protein content and the protein economy of a production facility. Substrate protein content is relevant, but it is not the only factor to consider when determining whether a substrate is suitable for the growth of mealworm larvae. When considering the frass produced in the ECD values, the best results were detected for TP, BSG, HC, and RB. Average values for WM and WB, whereas the worst results were accounted for by TC and DGM.

Pearson’s correlation coefficient showed that the amount of frass correlated well with the total amount of food supplied and consumed (r = 0.76 and 0.90). However, this was mostly due to the carrot fraction; in fact, the correlation with the feed was very low (r = 0.22).

## 4. Discussion

The need for an effective, inexpensive source of nutrient-rich diets, particularly protein-rich diets, for livestock and fish feed has led to the testing of various matrices that can produce large amounts of biomass over time. For this purpose, the potential use of *T. molitor* larvae represents an intriguing alternative to fish-based feed. To breed insects for use in animal feed, it is necessary to determine the most suitable nutritional and economic diet strategy for them. The majority of insect species exhibit a high degree of dietary specialisation. Consequently, any discrepancies in their nutritional requirements must be examined quantitatively. Measuring intake is the key to quantitative studies that aim to determine the absolute requirements for dietary constituents. The reason for this is differences in food efficiency, which is measured by intake and growth [23]. Single-substrate diets made from agricultural by-products and waste, such as cereal-derived substrates (WB, wheat flour, oat, barley, RB, etc.) with varying levels of protein, carbohydrates, fats, and fibre, have been proposed [36]. Due to good larval tolerability and a balanced nutritional profile, WB is the most widely used substrate and is often employed as a positive control in comparative studies. In addition, some trials have reported the exploitation and characterization of wheat products such as bread, bread remains, and cookies [36]. Additionally, Lienhard et al. investigated twenty-nine different substrates; among these, HC was also evaluated as a potential substrate for rearing *T. molitor* larvae [21]. By-products from the beverage industry have been tested worldwide, particularly BSG [36], whereas only a few studies have explored the rearing of *T. molitor* on mixtures of these substrates [11,12,13].

Studies on self-selection carried out on *Acheta domesticus* L., the house cricket, showed that these insects are capable of selecting nutrients [36]. The self-selection method proposed by Waldbauer and Friedman states that the search for optimal diets from multiple ingredients should allow insects to select the optimal ratios of each ingredient from the feed [35]. Recent studies have evaluated the attitude of *T. molitor* larvae in selecting nutrients, with the aim of improving understanding of their dietary requirements. These studies showed a high level of self-selection when the rearing substrate consists of a mixture of different raw materials. The *T. molitor* larvae selected the most favourable combination of macronutrients, promoting optimal growth even when the overall macronutrient composition of the substrate was unbalanced [36,37,38,39].

This selective feeding behaviour appears to be aimed at maximizing the intake of digestible energy (mainly from sugars and fats) and amino acids (from proteins), while minimizing the ingestion and metabolic costs associated with fibre degradation [37,38]. In addition, these data support the hypothesis that larvae actively avoid fibre-rich components during feeding, thereby improving feed conversion efficiency despite the heterogeneous composition of the rearing substrate. In carbohydrate-rich substrates, larvae primarily obtain glucose from starch, which is more readily digestible, while the fibrous fraction accumulates largely in the frass. By contrast, in extremely low-carbohydrate, high-fibre matrices rich in protein and fat, larvae appear to be able to partially exploit the fibrous fraction as an alternative energy source [39,40]. Hemicellulose is a mixture of polysaccharides and is associated with cellulose in plant cell walls [41]. The major polysaccharides in the hemicellulose fraction are based on β-l,4-1inked D-glycan backbones composed of glucose, xylose, mannose, and mannose and glucose (glucomannans units) [41]. Terra et al. reported the ability of insects to digest hemicellulose using hemicellulases [42]. Wang et al. reached the same conclusions by breeding larvae on corn stover [43]. These physiological adaptations underline the metabolic plasticity of *T. molitor*, which allows larvae to modulate nutrient assimilation pathways in response to substrate composition.

Mao et al. reported the breeding of *T. molitor* larvae on lignocellulosic waste matrices with the intent of degrading organic waste. The matrices were distillers’ grains (DG), and maize straw (MS). The study highlighted the capacity of *T. molitor* to degrade the fibrous fraction, leading to a decrease in the corresponding frass [40].

Mancini et al. reported on the larval growth performance of *T. molitor* reared on brewery spent grains, bread, and cookie leftovers. The results showed the influence of the chemical composition of the feeding substrates on the parameters investigated. In particular, it focuses on the growth speed and the amount of protein, carbohydrates, and lipids of the larvae. The results highlight the high plasticity of mealworm larvae and their potential to adapt to different substrates [44]. Baldacchino et al. studied the growth parameters using tomato pomace (TP) and bran-based feeds, such as brewer’s spent grain. The trials highlighted the influence on the speed of growth and the fatty acid composition of the larvae. Larval body composition partially reflected feed composition but remained within a narrow physiological range, consistent with previous reports [45]. These findings suggest that diet influences larval nutritional composition, though endogenous regulation maintains biochemical homeostasis within a limited species-specific range [25]. Fondevilla et al. investigated the impact of carbohydrate content and NDF on the performance of the larvae; three substrates were investigated, and the results showed no differences among trials on the growth performance and chemical composition of the larvae [46].

The amount of moisture contained in the substrate is considered a pivotal factor in the rearing phase, strongly influencing the life cycle parameters of larvae [21], which can tolerate extremely dry conditions for long periods, getting water both from the atmosphere and dried food. However, larval growth improves in humid environments (>70% RU) and in the presence of water sources, such as fresh vegetables [13].

In this study, eight different substrates were used in combination with carrots as a water source. The proximate composition of the substrates used in the trials reported in this paper showed comparable levels to those of other authors [45,46,47,48], except for NDF in DGM (higher in this study) and TP (higher in the other studies) [46].

Development times vary depending on the feed administered, temperature, and humidity [49]. In this experiment, the best conditions according to the literature were selected (25 °C and 60% RH). Each trial was carried out using a water source made from carrots. Therefore, development time was influenced mainly by substrate composition. In accordance with the research findings, trials conducted with WB and WM demonstrated the shortest development period. Consequently, WB is frequently employed as a control diet [25,45,46]. The development rate was found to be influenced by the protein content, with high protein feed showing shorter developing times [25,45]. In the experiment reported in this paper, HC had the highest protein values; however, larvae fed with this substrate showed similar development time to WB and WM; on the contrary, TC, which had a medium protein content, showed a significantly higher development time. This fact requires finding other reasons to explain the growth rates. Some authors correlate the growing speed with the vitamin content [36], starting from the self-selection capacity of the insects’ larvae. Moreover, the higher carbohydrate content of WM and WB could provide easily accessible energy during the growth stage. However, another fact to be considered is the amount of feed consumed. FCR showed similar results considering only the substrates; however, when the data from the water source (carrots) was implemented, it could be evinced that a significant difference was observed among TC and DGM, and the other substrates. These substrates showed minor efficiency in using the feed, which led to higher development times. High FCR data were positively correlated with high development times [25]. These data were in accordance with the residual and frass composition, which showed an inverse total N content with respect to the substrate (Figure 1). However, the larval protein content was statistically similar in all trials (Table 3), confirming the plasticity of *T. molitor* in relation to diet [44].

Feeding on a diet not sufficiently adapted to their needs means earlier pupation and lower weight gain. From this point of view, some authors reported that mealworms no longer gain weight in the last stages of development, preparing their bodies and reserves for the next development stage [50]. These assumptions agree with the results reported in this paper, where HC, WB, and WM showed the highest pupation rates and lower weight gain.

Substrates with lower levels of carbohydrates, such as BSG, TC, and DGM, had higher fibre content. The corresponding larvae showed different behaviour with the NDF fraction in the distribution of the hemicellulose and ADF fractions among larvae, residue, and frass.

Moreover, the excessive fibre levels of brewer’s spent grain (58.9%) and distilled grape marc (64.6%) could reduce digestibility and contribute to lower performance in larval growth, according to the studies of Ruschioni et al. and Morales-Ramos et al., who noted a negative effect on food conversion and weight when NDF was over 30% substrate composition [37,51]. In contrast, RB exhibited lower levels of NDF and unbalanced compositions (38.8% soluble carbohydrates and 15% lipids), which may explain the high mortality (52%) and poor conversion efficiency (3.23) observed. In addition, these effects may be attributed to low palatability or the presence of anti-nutritional compounds such as polyphenols [49].

Low-quality substrates require extended development and increased water input, which is met through carrot consumption. To maximize larval growth, carrots are often supplemented as an indispensable additional water supply [52,53]. Carrots may also provide compensatory nutrition due not only to their moisture content but also for simple sugars and vitamin content [48]. Growing performance is related to substrate composition with high carbohydrates and low proteins, resulting in larvae with better feed conversion and weight, according to literature data [20]. Weights of larvae and pupae, as well as frass production per unit of larval biomass, proved to be sensitive indicators of feed quality. These values reflect the nutritional balance and metabolic efficiency of the diet. The poorer the quality of the diet, the higher the frass values [25,53].

The present study promotes the use of agro-industrial by-products to reduce costs and environmental impact, excluding substrates with high mortality or low conversion (RB, TC, DGM) from the main formulations, and considering multi-substrate mixtures to optimize nutrients and reduce the risk of imbalances.

However, some questions remain unanswered or need to be verified on a case-by-case basis and require further consideration. For the most part, agro-industrial leftovers are a waste disposal expense for the business while being a zero-cost product for mealworm farming. However, the availability of the most effective by-products (HC, WB, and WM) on an industrial scale is linked to the presence of local processing companies. Brewer’s spent grain, tomato skins/pomace, and hemp press cake, along with the other by-products from the agro-industry, are often produced in relevant quantities and may generate disposal and logistics constraints for agro-industrial plants. In addition, the high moisture content and rapid spoilage of matrices, such as BSG and tomato residues, particularly increase handling costs when local collection or outlets are limited. The impact of contaminants or anti-nutritional factors on the quality of by-products (e.g., polyphenols in RB) should be evaluated on a case-by-case basis. Furthermore, seasonal and geographical variability must be considered when operating in standardized production.

From a cost-reduction perspective, the use of local agro-industrial by-products and residues appears to be a feasible and sustainable strategy for producing larval biomass with high nutritional value. Their valorization through mass rearing of insect larvae appears economically convenient and environmentally sustainable, reducing disposal costs while converting low-value residues into higher-value biomass through a virtuous circular, local, short-supply-chain approach.

Moreover, the transfer from laboratory-scale breeding to industrial-scale breeding involves a considerable increase in numbers. The problem addressed is reversed, and the simplest part turns out to be the supply of raw materials for feeding larvae. In contrast, a major technological upgrade is needed, starting with the recovery of eggs from adults, through to the breeding and recovery of larvae and processing for subsequent use in feed.

In addition, feed conversion efficiency under laboratory conditions should be confirmed at the industrial level.

## 5. Conclusions

The demand for animal protein is increasing. Insects are a sustainable source of protein and are reared on agro-industrial by-products to reduce costs and the environmental impact. The larvae of the yellow mealworm were reared on eight such products. These products include hemp cake and wheat bran. The experiment found that the larvae of *T. molitor* grew well, and the nutrition was suitable.

Hemp cake is the most efficient substrate, followed by wheat bran and wheat middling. Substrates with a high fibre content or unbalanced composition reduced growth and increased mortality. In addition, for the first time, this study carried out a full chemical characterization of the residual matrices after breeding and larval frass.

The larvae of *T. molitor* are a stable protein source, but variable for lipids and moisture. The substrates recommended are hemp cake, wheat bran, and wheat middling. Substrates that should be avoided include RB, TC, and DGM.

The study suggests that multi-substrate formulations can optimise nutrients and reduce imbalances.

Analysis performed on residual feed and insect frass supported the selective feeding behaviour and high plasticity of mealworm larvae and their potential to adapt to different substrates. Indeed, larvae try to maximize the intake of sugars, fats, and amino acids while minimizing the ingestion of fibre. However, under extreme conditions, larvae seem to be able to partially utilize the fibrous fraction as an alternative energy source.

Following the latest indications of the circular economy and according to this study, insects raised on suitable substrates have proven to be a valid alternative to fish meal and oil as the main ingredient in the production of feed for aquaculture.

The data reported were obtained under laboratory conditions. The next step for this research would be to extend to higher technological readiness levels (TRL 5–6); however, the results obtained are not necessarily directly translatable to industrial-scale production and need trials at an industrial level to be confirmed.

## Figures and Tables

**Table 1:** By-products used for larval rearing and their origin.

Substrate	Source
Wheat middling (WM)	Milling industry
Durum wheat bran (WB)	
Rice bran (RB)	
Hempseed cake (HC)	Agri-food industry
Tomato pomace (TP)	
Thistle cake (TC)	Cardoon seed oil extraction
Brewer’s spent grain (BSG)	Beverage industry
Distilled grape marc (DGM)	

**Table 2:** Proximate chemical composition of raw substrates (g/100 g ± RSD% *).

Substrates	Moisture	Ash	Tot. Lipids	Tot. Proteins	Carbohydrates	NDF
Tomato pomace (TP)	6.79 ± 4.98	5.13 ± 2.04	4.94 ± 2.95	13.65 ± 7.43	10.47 ± 2.73	58.68 ± 0.49
Brewer’s spent grain (BSG)	7.81 ± 9.12	3.36 ± 4.21	5.51 ± 4.40	18.63 ± 10.53	5.68 ± 12.35	58.86 ± 1.19
Hemp cake (HC)	8.15 ± 3.54	6.65 ± 4.34	11.25 ± 12.60	27.40 ± 4.86	10.95 ± 10.31	36.64 ± 2.82
Wheat middling (WM)	10.09 ± 7.47	3.87 ± 8.89	7.43 ± 3.92	15.77 ± 10.32	34.00 ± 0.19	34.53 ± 3.03
Durum wheat bran (WB)	10.10 ± 7.73	4.04 ± 3.76	7.37 ± 5.36	15.70 ± 10.79	34.00 ± 0.19	27.77 ± 0.21
Rice bran (RB)	7.34 ± 3.91	8.19 ± 2.29	15.43 ± 7.07	7.06 ± 12.38	38.79 ± 7.75	23.50 ± 12.80
Thistle cake (TC)	5.09 ± 2.84	5.20 ± 3.33	10.46 ± 2.58	17.03 ± 2.53	14.50 ± 3.95	47.15 ± 0.53
Distilled grape marc (DGM)	7.25 ± 2.01	5.27 ± 2.75	5.22 ± 5.07	10.07 ± 4.56	7.37 ± 3.28	64.57 ± 0.37

* RSD%: percentual relative standard deviation.

**Table 3:** Proximate chemical composition of larvae (g/100 g). Detailed statistical comparisons for each parameter are provided in Supplementary Table S1.

Substrate	Moisture	Ash	Total Lipids	Total Proteins	Carbohydrates
Tomato pomace (TP)	63.57 ^ab^*	1.16 ^ab^	12.94 ^a^	18.65 ^a^	4.42 ^ab^
Brewer’s spent grain (BSG)	61.32 ^b^	1.20 ^ab^	11.36 ^a^	18.65 ^a^	6.11 ^b^
Hemp cake (HC)	61.57 ^b^	1.10 ^ab^	11.60 ^a^	19.86 ^a^	5.12 ^b^
Wheat middling (WM)	61.06 ^b^	1.20 ^ab^	12.99 ^a^	19.04 ^a^	3.36 ^a^
Durum wheat bran (WB)	58.83 ^bc^	1.16 ^a^	14.00 ^a^	19.46 ^a^	6.17 ^b^
Rice bran (RB)	62.54 ^ab^	1.46 ^b^	12.36 ^a^	17.91 ^a^	5.76 ^b^
Thistle cake (TC)	65.66 ^a^	1.19 ^ab^	9.96 ^a^	19.67 ^a^	3.51 ^a^
Distilled grape marc (DGM)	59.49 ^c^	1.66 ^c^	16.70 ^b^	18.80 ^a^	3.94 ^a^

* Different letters superimposed on the numbers in the columns denote statistical disparities for *p* < 0.05.

**Table 4:** Developmental performance parameters of *Tenebrio molitor* larvae (means of 6 replicates) reared on eight agro-industrial by-products. Detailed statistical comparisons for each parameter are provided in Supplementary Table S1.

	Start (T_0_)	Harvest Time (T_end_)					
Substrates	n. Eggs/Pot	LD	LW	PW	TB	LM	PR
		n. Days	Grams			%	
Tomato pomace (TP)	217 ^a^*	113.7 ^a^	0.120 ^a^	0.119 ^ab^	18.96 ^ac^	25.87 ^a^	18.47 ^ab^
Brewer’s spent grain (BSG)	222 ^a^	107.0 ^ab^	0.117 ^a^	0.118 ^ab^	17.47 ^ac^	32.22 ^ae^	23.12 ^ab^
Hemp cake (HC)	236 ^a^	101.0 ^b^	0.136 ^b^	0.111 ^ab^	26.73 ^ab^	9.46 ^bc^	36.90 ^a^
Wheat middling (WM)	227 ^a^	97.3 ^b^	0.143 ^b^	0.121 ^a^	25.23 ^abc^	19.86 ^ab^	28.28 ^ab^
Durum wheat bran (WB)	241 ^a^	98.7 ^b^	0.135 ^b^	0.113 ^ab^	30.06 ^b^	4.27 ^c^	22.87 ^ab^
Rice bran (RB)	224 ^a^	133.2 ^c^	0.144 ^b^	0.088 ^bc^	13.21 ^c^	51.57 ^de^	20.74 ^ab^
Thistle cake (TC)	285 ^a^	142.7 ^c^	0.102 ^a^	0.098 ^abc^	22.05 ^abc^	21.57 ^ab^	15.89 ^ab^
Distilled grape marc (DGM)	229 ^a^	173.2 ^c^	0.098 ^a^	0.083 ^c^	12.70 ^c^	42.23 ^e^	10.93 ^b^

LD: larval development; LW: fresh larval weight; PW: fresh pupae weight; TB: total insect biomass; LM: larval mortality; PR: pupation rate. * Differentletters superimposed on the numbers in the columns denote statistical disparities for *p* < 0.05.

**Table 5:** Feed efficiency values of *Tenebrio molitor* larvae (means of 6 replicates) reared on different substrates. Detailed statistical comparisons for each parameter are provided in Supplementary Table S1.

Substrate	FCR	FCR_C_	FCR_Tot_	FP	ECI	PER	ECD
Tomato pomace (TP)	2.36 ^a^*	3.31 ^a^	5.67 ^a^	1.84 ^a^	0.42 ^a^	0.48 ^a^	0.24 ^a^
Brewer’s spent grain (BSG)	2.43 ^a^	3.92 ^a^	6.35 ^a^	1.67 ^a^	0.41 ^a^	0.30 ^b^	0.22 ^a^
Hemp cake (HC)	1.92 ^b^	5.15 ^b^	7.07 ^b^	1.42 ^b^	0.52 ^b^	0.35 ^b^	0.19 ^a^
Wheat middling (WM)	2.15 ^ce^	7.21 ^ac^	9.36 ^c^	1.15 ^c^	0.47 ^ab^	1.09 ^c^	0.14 ^b^
Durum wheat bran (WB)	2.17 ^c^	7.42 ^c^	9.58 ^bc^	1.18 ^c^	0.46 ^a^	0.83 ^c^	0.13 ^b^
Rice bran (RB)	3.23 ^de^	4.91 ^d^	8.14 ^d^	1.79 ^ad^	0.32 ^c^	0.70 ^c^	0.17 ^ab^
Thistle cake (TC)	2.44 ^e^	14.94 ^d^	17.38 ^ed^	2.50 ^d^	0.42 ^a^	0.59 ^a^	0.07 ^c^
Distilled grape marc (DGM)	2.74 ^de^	17.81 ^ed^	20.55 ^e^	2.49 ^ed^	0.37 ^c^	0.44 ^a^	0.06 ^c^

FCR: feed conversion ratio; FCR_C_: carrot consumption ratio; FCR_Tot_: feed conversion factor considering the total feed ingested; FP: frass production; ECI: efficiency of conversion of ingested food; PER: protein efficiency ratio; ECD: efficiency of conversion of digested food. * Differentletters superimposed on the numbers in the columns denote statistical disparities for *p* < 0.05.

## Data Availability

The original contributions presented in the study are included in the article/Supplementary Materials. Further inquiries can be directed to the corresponding authors.

## Supplementary Materials

The following supporting information can be downloaded at https://www.mdpi.com/article/10.3390/foods15020393/s1: Table S1: *p*-values for the comparison of the chemical composition of the biomass of larvae reared on different substrates. Mean differences were evaluated using multiple *t*-tests and controlled for multiple comparisons by the false discovery rate (FDR) approach (Benjamini, Krieger, and Yekutieli method), with the desired FDR (Q) set at 5%.; Table S2: *p*-values for the comparison between the chemical composition of raw substrates, remaining feed after consumption, and insect frass. Mean differences were evaluated using multiple *t*-tests and controlled for multiple comparisons by the false discovery rate (FDR) approach (Benjamini, Krieger, and Yekutieli method), with the desired FDR (Q) set at 5%.; Table S3: *p*-values for the comparison of developmental performance parameters of *Tenebrio molitor* larvae reared on eight agro-industrial by-products. Mean differences were evaluated using multiple t-tests and controlled for multiple comparisons by the false discovery rate (FDR) approach (Benjamini, Krieger, and Yekutieli method), with the desired FDR (Q) set at 5%.
