# Supplementary material for: Growth Performances and Nutritional Values of Tenebrio molitor Larvae: Influence of Different Agro-Industrial By-Product Diets
Source: Foods. 2026 Jan 22;15(2):393. doi: 10.3390/foods15020393 (PMC12840792; doi:10.3390/foods15020393)
Supplement: Supplementary file 1 [file foods-15-00393-s001.zip › foods-4109769-supplementary.pdf]

Table S1. *p*-values for the comparison of the chemical composition of the biomass of larvae reared on different substrates. Mean differences were evaluated using multiple *t*-tests and controlled for multiple comparisons by the False Discovery Rate (FDR) approach (Benjamini, Krieger, and Yekutieli method), with the desired FDR (*Q*) set at 5%.

Comparison	Humidity	Ash	Lipids	Proteins	Carbohydrates
**TP *vs.* BSG**	> 0.05	> 0.05	> 0.05	> 0.05	> 0.05
**TP *vs.* HC**	> 0.05	> 0.05	> 0.05	> 0.05	> 0.05
**TP *vs.* WM**	> 0.05	> 0.05	> 0.05	> 0.05	> 0.05
**TP *vs.* WB**	> 0.05	> 0.05	> 0.05	> 0.05	> 0.05
**TP *vs.* RB**	> 0.05	> 0.05	> 0.05	> 0.05	> 0.05
**TP *vs.* TC**	> 0.05	> 0.05	> 0.05	> 0.05	> 0.05
**TP *vs.* DGM**	**< 0.05**	**< 0.05**	**< 0.05**	> 0.05	> 0.05
**BSG *vs.* HC**	> 0.05	> 0.05	> 0.05	> 0.05	> 0.05
**BSG *vs.* WM**	> 0.05	> 0.05	> 0.05	> 0.05	> 0.05
**BSG *vs.* WB**	> 0.05	> 0.05	> 0.05	> 0.05	> 0.05
**BSG *vs.* RB**	> 0.05	> 0.05	> 0.05	> 0.05	> 0.05
**BSG *vs.* TC**	> 0.05	> 0.05	> 0.05	> 0.05	> 0.05
**BSG *vs.* DGM**	> 0.05	**< 0.05**	**< 0.05**	> 0.05	> 0.05
**HC *vs.* WM**	> 0.05	> 0.05	> 0.05	> 0.05	> 0.05
**HC *vs.* WB**	> 0.05	> 0.05	> 0.05	> 0.05	> 0.05
**HC *vs.* RB**	> 0.05	> 0.05	> 0.05	> 0.05	> 0.05
**HC *vs.* TC**	> 0.05	> 0.05	> 0.05	> 0.05	> 0.05
**HC *vs.* DGM**	< 0.05	**< 0.05**	**< 0.05**	> 0.05	> 0.05
**WM *vs.* WB**	< 0.05	> 0.05	> 0.05	> 0.05	**< 0.05**
**WM *vs.* RB**	> 0.05	> 0.05	> 0.05	> 0.05	> 0.05
**WM *vs.* TC**	> 0.05	> 0.05	> 0.05	> 0.05	> 0.05
**WM *vs.* DGM**	**< 0.05**	**< 0.05**	**< 0.05**	> 0.05	> 0.05
**WB *vs.* RB**	**< 0.05**	**< 0.05**	> 0.05	> 0.05	> 0.05
**WB *vs.* TC**	> 0.05	> 0.05	> 0.05	> 0.05	> 0.05
**WB *vs.* DGM**	> 0.05	**< 0.05**	**< 0.05**	> 0.05	> 0.05
**RB *vs.* TC**	> 0.05	> 0.05	> 0.05	> 0.05	> 0.05
**RB *vs.* DGM**	**< 0.05**	> 0.05	**< 0.05**	> 0.05	> 0.05
**TC *vs.* DGM**	**< 0,05**	**< 0,05**	**< 0,05**	**< 0,05**	> 0,05

Table S2. p-values for the comparison between the chemical composition of raw substrates, remaining feed after consumption, and insect frass. Mean differences were evaluated using multiple t-tests and controlled for multiple comparisons by the False Discovery Rate (FDR) approach (Benjamini, Krieger, and Yekutieli method), with the desired FDR (Q) set at 5%.

Comparison	Humidity	Ash	Lipids	Proteins	Carbohydrates	NDF	Hemicellulose	Cellulose	Lignin
**TP *vs.* TPR**	**< 0.05**	> 0.05	> 0.05	**< 0.05**	> 0.05	> 0.05	**< 0.05**	**< 0.05**	> 0.05
**TP *vs.* TPF**	**< 0.05**	**< 0.05**	**< 0.05**	> 0.05	> 0.05	**< 0.05**	**< 0.05**	**< 0.05**	**< 0.05**
**TPR *vs.* TPF**	**< 0.05**	**< 0.05**	**< 0.05**	**< 0.05**	> 0.05	**< 0.05**	**< 0.05**	**< 0.05**	**< 0.05**
**BSG *vs.* BSGR**	> 0.05	> 0.05	> 0.05	> 0.05	> 0.05	> 0.05	**< 0.05**	> 0.05	**< 0.05**
**BSG *vs.* BSGF**	< 0.05	< 0.05	**< 0.05**	> 0.05	< 0.05	**< 0.05**	**< 0.05**	> 0.05	**< 0.05**
**BSGR *vs.* BSGF**	< 0.05	> 0.05	**< 0.05**	> 0.05	> 0.05	**< 0.05**	< 0.05	**< 0.05**	**< 0.05**
**HC *vs.* HCR**	< 0.05	< 0.05	< 0.05	**< 0.05**	> 0.05	**< 0.05**	< 0.05	**< 0.05**	**< 0.05**
**HC *vs.* HCF**	< 0.05	**< 0.05**	> 0.05	< 0.05	**< 0.05**	> 0.05	< 0.05	> 0.05	> 0.05
**HCR *vs.* HCF**	**< 0.05**	**< 0.05**	> 0.05	**< 0.05**	**< 0.05**	**< 0.05**	> 0.05	**< 0.05**	**< 0.05**
**WM *vs.* WMR**	> 0.05	> 0.05	**< 0.05**	> 0.05	**< 0.05**	**< 0.05**	**< 0.05**	**< 0.05**	**< 0.05**
**WM *vs.* WMF**	> 0.05	**< 0.05**	**< 0.05**	> 0.05	**< 0.05**	**< 0.05**	**< 0.05**	**< 0.05**	**< 0.05**
**WMR *vs.* WMF**	> 0.05	**< 0.05**	> 0.05	> 0.05	> 0.05	> 0.05	> 0.05	**< 0.05**	**< 0.05**
**WB *vs.* WBR**	> 0.05	> 0.05	**< 0.05**	> 0.05	> 0.05	> 0.05	> 0.05	< 0.05	**< 0.05**
**WB *vs.* WBF**	> 0.05	> 0.05	**< 0.05**	> 0.05	**< 0.05**	**< 0.05**	> 0.05	**< 0.05**	**< 0.05**
**WBR *vs.* WBF**	> 0.05	> 0.05	> 0.05	> 0.05	**< 0.05**	**< 0.05**	**< 0.05**	**< 0.05**	**< 0.05**
**RB *vs.* RBR**	**< 0.05**	**< 0.05**	**< 0.05**	**< 0.05**	> 0.05	> 0.05	**< 0.05**	**< 0.05**	**< 0.05**
**RB *vs.* RBF**	**< 0.05**	**< 0.05**	> 0.05	< 0.05	**< 0.05**	**< 0.05**	**< 0.05**	**< 0.05**	**< 0.05**
**RBR *vs.* RBF**	> 0.05	**< 0.05**	> 0.05	> 0.05	**< 0.05**	**< 0.05**	**< 0.05**	> 0.05	**< 0.05**
**TC *vs.* TCR**	**< 0.05**	**< 0.05**	**< 0.05**	**< 0.05**	**< 0.05**	**< 0.05**	**< 0.05**	**< 0.05**	**< 0.05**
**TC *vs.* TCF**	**< 0.05**	**< 0.05**	> 0.05	**< 0.05**	> 0.05	**< 0.05**	**< 0.05**	> 0.05	**< 0.05**
**TCR *vs.* TCF**	**< 0.05**	**< 0.05**	> 0.05	**< 0.05**	> 0.05	**< 0.05**	**< 0.05**	**< 0.05**	**< 0.05**
**DGM *vs.* DGMR**	**< 0.05**	> 0.05	> 0.05	> 0.05	**< 0.05**	**< 0.05**	> 0.05	**< 0.05**	< 0.05
**DGM *vs.* DGMF**	**< 0.05**	**< 0.05**	> 0.05	**< 0.05**	> 0.05	**< 0.05**	> 0.05	**< 0.05**	**< 0.05**
**DGMR *vs.* DGMF**	> 0.05	**< 0.05**	**< 0.05**	**< 0.05**	**< 0.05**	**< 0.05**	> 0.05	**< 0.05**	**< 0.05**

Table S3. *p*-values for the comparison of developmental performance parameters of *Tenebrio molitor* larvae reared on eight agro-industrial by-products. Mean differences were evaluated using multiple *t*-tests and controlled for multiple comparisons by the False Discovery Rate (FDR) approach (Benjamini, Krieger, and Yekutieli method), with the desired FDR (*Q*) set at 5%.

**Comparison**	**LD n. days**	**Eggs/pot**	**Larval weight**	**Pupal weight**	**Insect biomass**	**Mortality**	**Pupation**	**FP**	**FCR**	**CCR**	**ECI**	**ECI**	**ECD**
**TP *vs.* BSG**	> 0.05	> 0.05	> 0.05	> 0.05	> 0.05	> 0.05	> 0.05	> 0.05	> 0.05	> 0.05	> 0.05	> 0.05	> 0.05
**TP *vs.* HC**	**< 0.05**	> 0.05	**< 0.05**	**< 0.05**	**< 0.05**	**< 0.05**	**< 0.05**	**< 0.05**	**< 0.05**	**< 0.05**	**< 0.05**	**< 0.05**	**< 0.05**
**TP *vs.* WM**	**< 0.05**	> 0.05	**< 0.05**	> 0.05	> 0.05	> 0.05	> 0.05	**< 0.05**	**< 0.05**	**< 0.05**	**< 0.05**	> 0.05	**< 0.05**
**TP *vs.* WB**	**< 0.05**	> 0.05	**< 0.05**	**< 0.05**	**< 0.05**	**< 0.05**	> 0.05	**< 0.05**	**< 0.05**	**< 0.05**	> 0.05	**< 0.05**	**< 0.05**
**TP *vs.* RB**	**< 0.05**	> 0.05	> 0.05	< 0.05	< 0.05	**< 0.05**	> 0.05	> 0.05	**< 0.05**	**< 0.05**	**< 0.05**	< 0.05	**< 0.05**
**TP *vs.* TC**	**< 0.05**	> 0.05	**< 0.05**	**< 0.05**	> 0.05	> 0.05	> 0.05	**< 0.05**	> 0.05	**< 0.05**	**< 0.05**	**< 0.05**	**< 0.05**
**TP *vs.* DGM**	**< 0.05**	> 0.05	**< 0.05**	**< 0.05**	**< 0.05**	**< 0.05**	**< 0.05**	**< 0.05**	**< 0.05**	**< 0.05**	**< 0.05**	**< 0.05**	**< 0.05**
**BSG *vs.* HC**	< 0.05	> 0.05	**< 0.05**	< 0.05	< 0.05	**< 0.05**	> 0.05	**< 0.05**	< 0.05	< 0.05	**< 0.05**	**< 0.05**	**< 0.05**
**BSG *vs.* WM**	< 0.05	> 0.05	**< 0.05**	> 0.05	> 0.05	**< 0.05**	> 0.05	**< 0.05**	**< 0.05**	**< 0.05**	> 0.05	> 0.05	**< 0.05**
**BSG *vs.* WB**	**< 0.05**	> 0.05	**< 0.05**	> 0.05	**< 0.05**	**< 0.05**	> 0.05	**< 0.05**	**< 0.05**	**< 0.05**	**< 0.05**	**< 0.05**	**< 0.05**
**BSG *vs.* RB**	**< 0.05**	> 0.05	> 0.05	**< 0.05**	> 0.05	**< 0.05**	> 0.05	> 0.05	**< 0.05**	**< 0.05**	**< 0.05**	**< 0.05**	**< 0.05**
**BSG *vs.* TC**	**< 0.05**	> 0.05	**< 0.05**	**< 0.05**	> 0.05	**< 0.05**	> 0.05	**< 0.05**	> 0.05	**< 0.05**	> 0.05	**< 0.05**	**< 0.05**
**BSG *vs.* DGM**	**< 0.05**	> 0.05	> 0.05	**< 0.05**	> 0.05	< 0.05	< 0.05	**< 0.05**	< 0.05	**< 0.05**	> 0.05	**< 0.05**	**< 0.05**
**HC *vs.* WM**	> 0.05	> 0.05	> 0.05	**< 0.05**	> 0.05	> 0.05	> 0.05	**< 0.05**	**< 0.05**	> 0.05	**< 0.05**	> 0.05	**< 0.05**
**HC *vs.* WB**	> 0.05	> 0.05	> 0.05	> 0.05	> 0.05	> 0.05	> 0.05	**< 0.05**	**< 0.05**	**< 0.05**	**< 0.05**	> 0.05	**< 0.05**
**HC *vs.* RB**	**< 0.05**	> 0.05	> 0.05	< 0.05	**< 0.05**	**< 0.05**	> 0.05	< 0.05	**< 0.05**	> 0.05	**< 0.05**	**< 0.05**	**< 0.05**
**HC *vs.* TC**	**< 0.05**	> 0.05	**< 0.05**	> 0.05	> 0.05	**< 0.05**	**< 0.05**	**< 0.05**	**< 0.05**	**< 0.05**	**< 0.05**	**< 0.05**	**< 0.05**
**HC *vs.* DGM**	**< 0.05**	> 0.05	**< 0.05**	**< 0.05**	**< 0.05**	**< 0.05**	**< 0.05**	**< 0.05**	**< 0.05**	**< 0.05**	**< 0.05**	**< 0.05**	**< 0.05**
**WM *vs.* WB**	> 0.05	> 0.05	> 0.05	> 0.05	> 0.05	> 0.05	> 0.05	> 0.05	> 0.05	> 0.05	> 0.05	> 0.05	> 0.05
**WM *vs.* RB**	**< 0.05**	> 0.05	> 0.05	**< 0.05**	**< 0.05**	**< 0.05**	> 0.05	**< 0.05**	**< 0.05**	> 0.05	**< 0.05**	**< 0.05**	> 0.05
**WM *vs.* TC**	**< 0.05**	> 0.05	**< 0.05**	**< 0.05**	> 0.05	> 0.05	> 0.05	**< 0.05**	> 0.05	**< 0.05**	> 0.05	**< 0.05**	> 0.05
**WM *vs.* DGM**	**< 0.05**	> 0.05	**< 0.05**	**< 0.05**	**< 0.05**	**< 0.05**	**< 0.05**	**< 0.05**	> 0.05	**< 0.05**	> 0.05	**< 0.05**	> 0.05
**WB *vs.* RB**	**< 0.05**	> 0.05	> 0.05	**< 0.05**	**< 0.05**	**< 0.05**	> 0.05	**< 0.05**	**< 0.05**	**< 0.05**	**< 0.05**	**< 0.05**	**< 0.05**
**WB *vs.* TC**	**< 0.05**	> 0.05	**< 0.05**	**< 0.05**	**< 0.05**	**< 0.05**	**< 0.05**	**< 0.05**	> 0.05	**< 0.05**	**< 0.05**	**< 0.05**	**< 0.05**
**WB *vs.* DGM**	**< 0.05**	> 0.05	**< 0.05**	**< 0.05**	**< 0.05**	**< 0.05**	**< 0.05**	**< 0.05**	> 0.05	**< 0.05**	**< 0.05**	**< 0.05**	**< 0.05**
**RB *vs.* TC**	**< 0.05**	> 0.05	> 0.05	> 0.05	**< 0.05**	**< 0.05**	> 0.05	**< 0.05**	**< 0.05**	**< 0.05**	> 0.05	> 0.05	> 0.05
**RB *vs.* DGM**	**< 0.05**	> 0.05	> 0.05	> 0.05	> 0.05	> 0.05	> 0.05	**< 0.05**	> 0.05	**< 0.05**	**< 0.05**	> 0.05	> 0.05
**TC *vs.* DGM**	> 0.05	> 0.05	> 0.05	> 0.05	> 0.05	**< 0.05**	> 0.05	> 0.05	> 0.05	> 0.05	> 0.05	> 0.05	> 0.05
